# Translation between the Neer- and the AO/OTA-classification for proximal humeral fractures: do we need to be bilingual to interpret the scientific literature?

**DOI:** 10.1186/1756-0500-6-69

**Published:** 2013-02-25

**Authors:** Stig Brorson, Henrik Eckardt, Laurent Audigé, Bernd Rolauffs, Christian Bahrs

**Affiliations:** 1 Department of Orthopaedic Surgery, Herlev University Hospital, Herlev, Denmark; 2 Department of Orthopaedic Surgery, Rigshospitalet, Copenhagen, Denmark; 3 Schulthess Klinik, Zürich, Switzerland; 4 Clinic for Traumatology and Reconstructive Surgery, Berufsgenossenschaftliche Unfallklinik Tübingen, Eberhard-Karls University Tübingen, Tübingen, Germany

**Keywords:** Proximal humeral fractures, Proximal humerus fractures, Shoulder fractures, Fracture classification, Neer, AO, OTA

## Abstract

**Background:**

The reporting and interpretation of data from clinical trials of proximal humeral fractures are hampered by the use of two partly incommensurable fracture classification systems: the Neer classification and the AO/OTA classification. It remains difficult to interpret and generalize results, to conduct prognostic studies, and to obtain consensus on treatment recommendations when concise definitions and a common ‘fracture language’ are lacking. Thus, we compared both classifications systems using primary data from large clinical studies to assess how thoroughly both systems conveyed clinically important classification information.

**Methods:**

Classification data from each study were organized in a cross-table covering the 432 theoretically possible combinations between the 16 Neer categories and the 27 AO/OTA subgroups, and the plausibility of all observed combinations were assessed and discussed by the authors until consensus.

**Results:**

We analyzed primary data from 2530 observations from seven studies providing primary data from both classification systems. Thirty-five percent (151 out of 432) of the combinations were considered ‘not plausible’ and thirty-four percent (149 out of 432) were considered ‘problematic’.

**Conclusions:**

Clinically important information was lost within both classification systems. Most important, the varus/valgus distinction was not found within the Neer classification and a clear definition of displacement was lacking in the AO/OTA classification. We encourage surgeons and researches to report data from both classification systems for a more thorough description of the fracture patterns and to enable cross-checking of the coding. A suitable table for cross-checking of the coding is provided herein.

## Background

Within the last decades there has been a quest for randomised trials, well-conducted observational studies, and systematic reviews of interventions for fractures of the proximal humerus. Systematic reviews have been inconclusive [1-6] and evidence based recommendations are lacking.

The performance of randomised clinical trials usually involves multiple centres to gain sufficient statistical power, especially in complex fracture patterns [7-10]. The performance and interpretation of multi-centre trials are facilitated by a rigorous approach to classification defining the study population prior to clinical interventions. The reporting and interpretation of data from clinical trials of proximal humeral fractures are hampered by the use of two partly incommensurable fracture classification systems. It remains difficult to interpret and generalize results, to conduct prognostic studies, and to obtain consensus on treatment recommendations when concise definitions and a common ‘fracture language’ are lacking.

**Figure 1:**
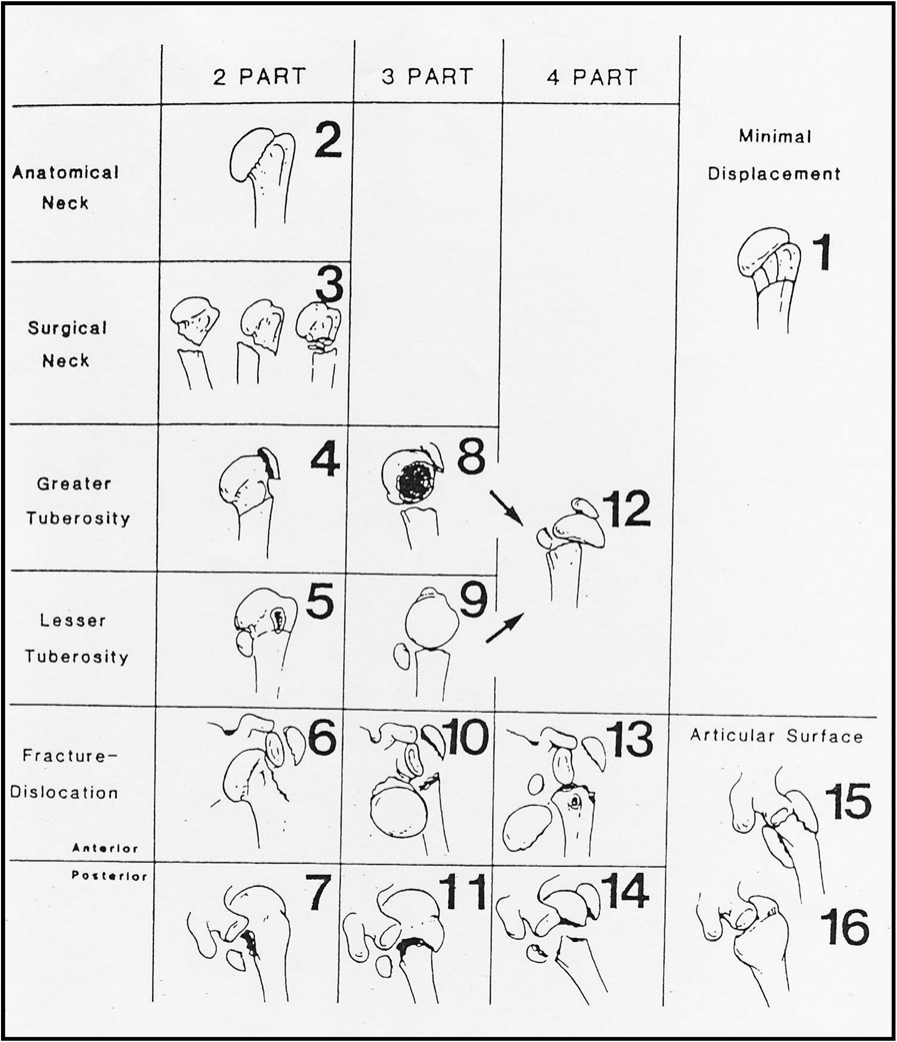
**The 16 categories of the Neer classification. **A fracture is considered displaced if one or more of the four segments are displaced more than 1 centimeter or angulated more than 45°. Modified from Neer 1970 [11] with permission from JBJS Am, Rockwater Inc.

**Figure 2:**
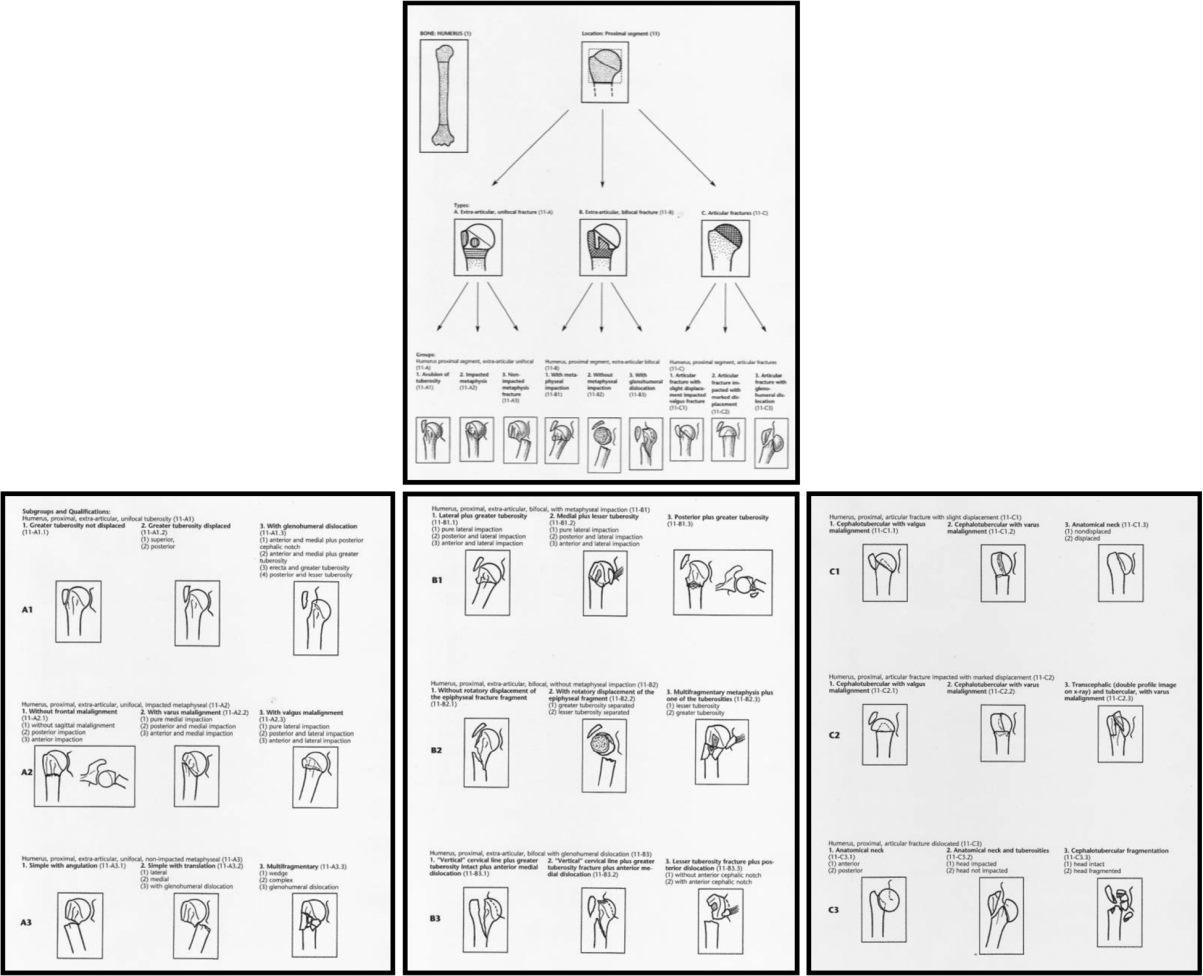
**The 3 types, 9 groups, and 27 subgroups of the AO/OTA classification. **Reprinted from. Marsh 2007 [14] with permission from JOT, Copyright Clearance Center.

The two classification systems most frequently used in the scientific literature are the classifications proposed by Charles Neer in 1970 (Figure 1) [11], updated in 2002 [12], and the AO/OTA classification, based on the Müller classification from 1990 [13], and updated in 2007 (Figure 2) [14]. To our knowledge, the translation problems between these classification systems have not been systematically studied. Further, the assumption that type A are 2- part fractures, type B are 3-part fractures, and type C are 4-part fractures is commonly held [15-17].

We aimed to systematically search and to analyze large clinical studies reporting data within both classification systems. Subsequently, we defined the terms ‘plausible’, ‘problematic’, and ‘not plausible’, and discussed all observed combinations between the two classification systems accordingly. Finally, we proposed a cross-table for use in the scientific literature containing all combinations of Neer categories and AO/OTA subgroups.

## Methods

We searched Pubmed, Embase, Cochrane Library, and Web of Science (2001 to June 2012) to identify large clinical studies classifying displaced fractures of the proximal humeral fractures according to both classification systems.

### Search strategy

((((humer* OR shoulder*) AND (break* OR broken* OR fractu*)) OR (“Shoulder Fractures”[Mesh] OR “Humeral Fractures”[Mesh])) AND (AO class* OR (AO system*) OR AO found* OR ASIF* OR OTA* OR Müller* OR Mueller* OR arbeitsgemeinschaft* OR osteosyntesefragen*)) AND (Neer*)).

We included studies with more than 100 fractures of the proximal humerus published within the last ten years containing data from both classification systems. Authors from all studies were contacted by e-mail for unpublished primary classification data (Additional file 1).

One reviewer (SB) conducted the literature search and identified studies that were clearly not relevant. The full text of potentially eligible studies was independently assessed by two reviewers (SB and HE). Disagreements were resolved by discussion. Classification data from each study were organized in a cross-table covering all 432 theoretical combinations between the 16 Neer categories and the 27 AO/OTA subgroups.

We further assessed the ‘plausibility’ of all combinations appearing in our data. Clearly possible combinations, for example, greater tuberosity fracture-dislocations in Neer (category 6) and in AO/OTA (subgroup A1.3) were termed ‘plausible’. Clearly impossible combinations, like articular surface fractures in Neer (categories 15 and 16) and extra-articular fracture in AO/OTA (type A or B), were termed ‘not plausible’. Other combinations which could not clearly be ruled out were termed ‘problematic’ and discussed further in the manuscript. A priori, we assumed a common understanding within the two classification systems of regarding whether a fracture was also dislocated.

## Results

We identified eleven [18-28] studies with more than 100 fractures classified according to both classification systems (Table 1). No individual classification data could be extracted from published data. The observed combinations were qualitatively reported in one study [24]. Authors from nine studies responded and authors from seven studies provided primary data on classification. We included all consecutive patients presenting a proximal humeral fracture. Only three studies [18,19,22] included unselected patients. The remaining studies selected patients according to image quality or to a specific treatment modality (Additional file 2).

**Table 1:** Studies with more than 100 proximal humeral fractures classified according to both the AO/OTA- and the Neer-classification

**Study**	**Fractures (n)**	**Included fractures**	**Setting**	**Data obtained**
Bahrs^18,19^	780	All fracture patterns	Register	yes
Bartsch^20^	102	Neer IV, V, and VI	Locking plate osteosynthesis	no
Kettler^26^	255	Displaced fractures	Locking plate osteosynthesis	yes
Hirschmann^25^	119	Displaced fractures	Locking plate osteosynthesis	yes
Solberg^28^	122	Three- and four-part fractures	Locking plate versus HA	yes
Brunner^21^	158	Displaced fractures	Locking plate osteosynthesis	no
Dietrich^23^	111	Three- and four-part fractures	Locking plate versus HA	no
Pelegri^27^	252	All fracture patterns	Register	no
Gumina^24^	227	Non-operatively treated	Observer study	yes
Court-Brown^22^	1027	All fracture patterns	Register	yes

**Figure 3:**
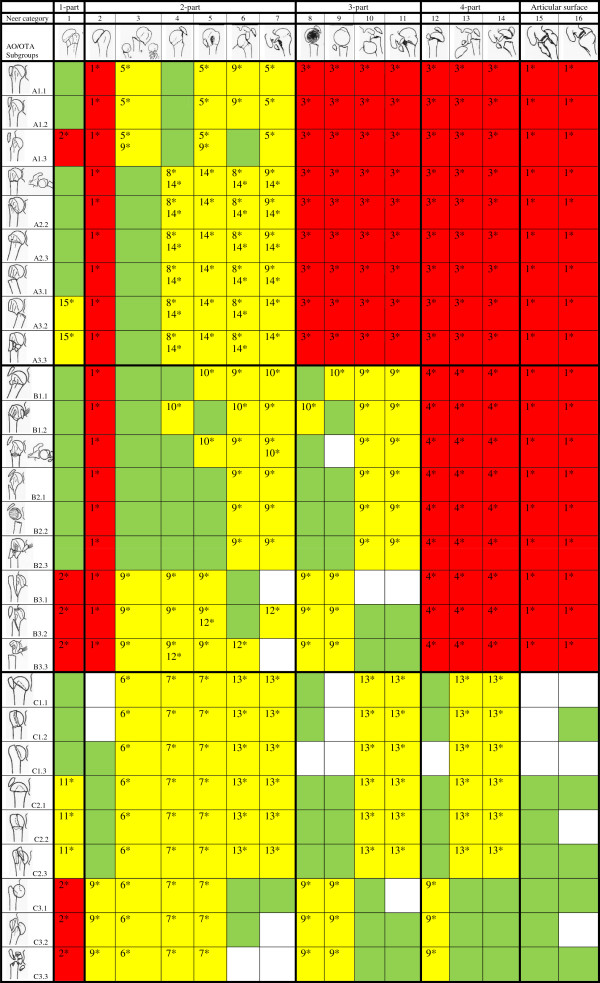
**Plausibility of combinations between the Neer- and the AO/OTA-classification. **Combinations in green appear in our data and are considered ‘plausuble’. Combinations in white do not appear in our data. Combinations in red are considered ‘not plausible’. Combinations in yellow are considered ‘problematic’. Pictograms modified from Marsh JL et al. [14] and Neer [11].

Thirty seven percent (158 out of 432) of the theoretically possible combinations between the two classification systems were used at least once (Additional file 3). Eleven percent of these combinations (18 out of 158) were considered ‘not-plausible’, and twenty-eight percent (45 out of 158) were considered ‘problematic’ (Figure 3). The absolute numbers of each combination are given for the three studies using unselected cases (Additional file 3) .

Bahrs et al. [18,19] provided data from a database containing 780 unselected proximal humeral fractures. Fourteen percent (109 out of 780) of the fractures were classified as minimally displaced (Neer category 1). The fractures were classified according to the Neer- and the AO/OTA-classification by two experienced trauma surgeons with a special interest in proximal humeral fractures in a consensus conference [19]. Based on plain radiographs and CT-scans Kettler et al. [26] classified 225 displaced 2-, 3- and 4-part fractures (mean age 66) in patients treated with locking plates. In Hirschmann et al. [25] one author not involved in surgical procedures classified 119 2-, 3- and 4-part fractures (mean age 68) based on plain radiographs in patients treated with locking plates. Solberg et al. [28] classified 122 3-, and 4-part fractures (mean age 67) treated with locking plates or hemiarthroplasty based on plain radiographs or CT-scans. In Gumina et al. [24] two authors classified 227 fractures based on plain radiographs (mean age 73). Cases were selected according to quality of images. In Court-Brown et al. [22] 1,027 unselected proximal humeral fractures were classified by one author. 49% of the fractures were classified as non-displaced (mean age 66).

We did not find it meaningful to report the marginal distribution of all combinations because most studies selected their patients for specific purposes. For example, some studies included only patients treated with locking plates while other studies included 3- and 4-part fractures only (Table 1).

## Discussion

We analysed 2530 pairs of classification data on proximal humeral fractures classified according to the Neer- and the AO/OTA-classification. The plausibility of all combinations was discussed.

### ‘Not plausible’ combinations

Thirty-five percent (151 out of 432 combinations) were considered ‘not plausible’ (red boxes in Figure 3):

1* Intra-articular fractures cannot be extra-articular. Neer category 2 (anatomical neck), and Neer categories 15 and 16 (articular surface) cannot be AO/OTA type A or B

2* Fracture-dislocations cannot be minimally displaced: AO/OTA fracture-dislocations (subgroup A1.3, group B3 and C3) cannot appear as Neer category 1

3* AO/OTA type A fractures are unifocal and cannot appear as displaced 3- or 4-part fractures (Neer categories 8, 9, 10, 11, 12, 13, and 14)

4* AO/OTA type B fractures are bifocal and cannot appear as displaced 4-part fractures (Neer categories 12, 13, and 14)

### ‘Problematic’ combinations

Thirty-four percent (149 out of 432 combinations) were considered ‘problematic’ (yellow spaces in Figure 3):

5* A1 fractures are isolated greater tuberosity fractures. They cannot appear as Neer 2-part surgical neck fractures (Neer category 3) or isolated lesser tuberosity fractures (Neer categories 5 and 7)

6* Isolated surgical neck fractures (Neer category 3) cannot be intra-articular (AO/OTA type C) unless a minimally displaced intra-articular fracture is present

7* Isolated greater or lesser tuberosity fractures (Neer categories 4 and 5) cannot be intra-articular (AO/OTA type C) unless a minimally displaced intra-articular fracture is also present

8* Group A2 and A3 do not involve the greater tuberosity and cannot appear as isolated greater tuberosity fracture (Neer categories 4 and 6)

9* A fracture-dislocation in the Neer classification (categories 6, 7, 10, 11, 13, or 14) can only appear as a fracture-dislocation within the AO/OTA classification (subgroups A1.3, A3.2, A3.3, group B3, or C3)

10* Subgroups B1.1 and B1.3 involve the greater tuberosity but not the lesser tuberosity and can therefore not appear as Neer categories 5, 7, or 9. Similarly, subgroup B1.2 fractures only involve the lesser tuberosity and cannot appear as greater tuberosity fractures (Neer categories 4, 6, or 8)

11* In the AO/OTA classification ‘slight displacement’ (group C1) cannot clearly be distinguished from ‘marked displacement’ (group C2). However, ‘marked displaced’ fractures (group C2) should not appear as ‘minimally displaced’ fractures within the Neer classification (category 1)

12* Subgroup B3.2 fracture-dislocations involve the greater tuberosity but not the lesser and cannot appear as Neer category 5 or 7. Similarly, subgroup B3.3 lesser tuberosity fractures do not involve the greater tuberosity (Neer categories 4 and 6)

13* Group C1 and C2 fractures are not dislocated and can therefore not appear as Neer categories 6, 7, 10, 11, 13, and 14

14* Groups A2 and A3 fractures are unifocal, impacted, metaphyseal fractures and cannot appear as isolated, displaced greater or lesser tuberosity fractures (Neer categories 4, 5, 6, and 7)

15* Subgroups A3.2 and A3.3 should not be classified as ‘minimally displaced’ fractures within the Neer classification (category 1) as they are translated or multi-fragmentary, and thus unstable. Neer defined ‘minimally displaced’ fractures as stable

### AO/OTA type A-,B-, and C- fractures

The commonly held assumption that type A are 2- part fractures, type B are 3-part fractures, and type C are 4-part fractures [15-17] was not supported by our data. One-part fractures (Neer category 1) can correspond to at least 15 different AO/OTA subgroups, and ‘classical’ four-part fractures (Neer category 12) can be classified into at least 8 different AO/OTA subgroups.

In the latest version of the AO/OTA classification [14] it is stated that type B type fractures represent three-part fractures, or fracture-dislocations by the Neer classification. However, we found that type B patterns could also appear as Neer 1- and 2-part fractures. The B1.1 valgus impacted fracture is common (15%) [29], and unique to the AO/OTA classification in that the humeral head is not rotated. This fracture pattern may present as a 1-part, 2-part, or 3-part fracture within the Neer system.

In the original AO-classification the authors mention that ‘…in B1 and B2, the fracture lines involve only the very borders of the articular surface. Articular impairment is more severe in B3 fractures which should be considered as an intermediate pattern between type B and C fractures’ [13]. This opens for translating Neer four-part fractures into AO/OTA type B. However, in the original AO-classification only 2- and 3-part fractures are depicted in illustrations of group C1 and C2 fractures. We suggest that 4-part fractures should not be classified as extra-articular (type A or B), but this problem remains unsolved.

Type C fractures can appear as 1-, 2-, 3-, or 4-part fractures within the Neer classification. However, in C2 fractures there is no distinction between 3- and 4-part fractures. The valgus-impacted four-part fracture was initially unique to the AO classification (subgroups C1.1 and C2.1) [30,31] but it was included by Neer classification in the 2002 revision [12]. In this pattern the medial periosteum remains intact and the pattern has been considered a ‘precursor’ to the ‘classical’ four-part fracture (Neer category 12). Subgroups C1.1 and C2.1 fractures can appear as 1-part, 2-part, 3-part or 4-part fractures within the Neer classification.

### The varus/valgus distinction

The varus/valgus distinction is prognostically and therapeutically important and unique to the AO/OTA classification. Generally, varus displacement is considered prognostically worse than valgus displacement [32]. Valgus impacted fractures appear as AO/OTA subgroups A2.3, B1.1, C1.1, and C2.1 which cover 1-part, 2-part, 3-part, and 4-part patterns within the Neer classification. The varus impacted fracture patterns include subgroups A2.2, B1.2, C1.2, C2.2, and C2.3 covering 1-part, 2-part, 3-part and 4-part patterns within the Neer classification. However, with no clear definition of displacement and no guidelines for rotation in anterior-posterior radiographs, it remains difficult to distinguish valgus and varus patterns with metaphyseal impaction (subgroups B1.1 and B1.2) from ‘marked displaced’ and impacted valgus and varus patterns (subgroups C2.1 and C2.2).

### Neer 1-,2-,3-, and 4-part fractures

It is unclear whether displaced 2-part fractures (Neer categories 2, 3, 4, 5, 6, or 7) or 4-part fractures (Neer categories 12, 13, and 14) can appear as bifocal fracture patterns (AO/OTA type B). If fracture lines not defined as displaced within the Neer classification are present these combinations are possible.

Neer defined 3-part fractures (categories 8 and 9) as extra-articular [11]. However, in our material they also appear as intra-articular (type C) fractures. The level of fracture lines in Neer categories 8–12 is not specified, and it is not clear whether the surgical or the anatomical neck is involved in 3- and 4-part fractures.

Four-part patterns in the Neer system (categories 12, 13, and 14) do not correlate to specific sub-groups within the AO system. There is no indication of fracture level but extra-articular fractures (surgical neck) with displacement of both tuberosities may occur.

Neer categories 4 and 5 cannot be translated directly into a certain group or subgroup in the AO/OTA classification because displacement is not clearly defined within the AO/OTA classification. Further, it remains unclear how isolated lesser tuberosity fractures should be classified within the AO/OTA classification.

### Factors potentially affecting classification

Factors possibly affecting classification include imaging modality and quality. The classifications were originally developed based on conventional radiographs. To classify a fracture at least two views without osseous overlapping are needed. Certain fracture patterns may be detected better by certain modalities or views, for example, tuberosity fractures in axillary views or articular fractures in CT-scans.

Observer agreement within both classifications has been extensively studied within the last twenty years [33-35]. Most studies have reported low kappa-values for inter- and intra-observer agreement with no clinically significant improvement by adding high quality radiographs, supplemental views, CT- or 3D CT-scans, or by including only experienced observers. We are not aware of any study examining the consistency of patterns between the two systems. However, the lack of concise definitions may confound both the process of translation between the classification systems and the reliability within each classification system.

## Conclusions

Clinical important information is lost within both classification systems. Some fractures are best described in the AO/OTA classification some in the Neer classification. The clinically important varus/valgus distinction is not found within the Neer classification and a clear definition of displacement is lacking within the AO/OTA classification. We encourage surgeons and researchers to report data from both classification systems for a more thorough description of the fracture patterns and to enable cross-checking of the coding.

## Ethical approval

The study did not involve human individuals.

## Competing interests

The authors declare that they have no competing interests. SB was supported financially by Göran Bauer’s Grant, The Danish Rheumatism Association, and the Danish Agency for Science, Technology and Innovation.

## Authors’ contributions

SB conceived the idea and designed the study. SB and HE gathered the data. The data-analysis was performed by SB and CB. SB wrote the manuscript. All authors were involved in critical revision of the paper. All authors read and approved the final manuscript.

## Supplementary Material

Additional file 1 Flow chart. Click here for file

Additional file 2 PRISMA 2009 Checklist. Click here for file

Additional file 3 Observed combinations between the AO/OTA- and the Neer-classification in 2530 pairs of observations in seven studies. Click here for file
