# Supplementary material for: Translation between the Neer- and the AO/OTA-classification for proximal humeral fractures: do we need to be bilingual to interpret the scientific literature?
Source: BMC Res Notes. 2013 Feb 25;6:69. doi: 10.1186/1756-0500-6-69 (PMC3610277; doi:10.1186/1756-0500-6-69)
Supplement: Additional file 1 — Flow chart. [file 1756-0500-6-69-S1.doc]

Additional file 1

Records identified through database searching
(n = 25 )

Additional records identified through other sources
(n = 17 )

Records after duplicates removed
(n = 42 )

Records screened
(n = 42 )

Records excluded

Clearly not relevant (n=19)
(n = )

Full-text articles assessed for eligibility
(n = 23 )

Full-text articles excluded

< 100 patients (n =12 )

Contact to authors
(n = 11)

Studies included in quantitative synthesis
(n = 7)

No response (n=2)

No data provided (n = 2)

Flow chart
