# Supplementary material for: Translation between the Neer- and the AO/OTA-classification for proximal humeral fractures: do we need to be bilingual to interpret the scientific literature?
Source: BMC Res Notes. 2013 Feb 25;6:69. doi: 10.1186/1756-0500-6-69 (PMC3610277; doi:10.1186/1756-0500-6-69)
Supplement: Additional file 3 — Observed combinations between the AO/OTA- and the Neer-classification in 2530 pairs of observations in seven studies. [file 1756-0500-6-69-S3.doc]

Additional file 3

**Observed combinations between the AO/OTA- and the Neer-classification in 2530 pairs of observations in seven studies**

|  | 1-part | 2-part | | | | | | 3-part | | | | 4-part | | | Art. surface | |
| --- | --- | --- | --- | --- | --- | --- | --- | --- | --- | --- | --- | --- | --- | --- | --- | --- |
|  | 1 | 2 | 3 | 4 | 5 | 6 | 7 | 8 | 9 | 10 | 11 | 12 | 13 | 14 | 15 | 16 |
| A1.1 | 60 |  |  | 4 |  |  |  |  |  |  |  |  |  |  |  |  |
| A1.2 | 103 |  |  | 40 | 1 | 1 |  |  |  |  |  |  |  |  |  |  |
| A1.3 | 4 |  |  | 2 |  | 54 | 1 |  |  |  |  |  |  |  |  |  |
| A2.1 | 126 |  | 7 |  |  |  |  |  |  |  |  |  |  |  |  |  |
| A2.2 | 94 |  | 55 |  |  |  |  |  |  |  |  |  |  |  |  |  |
| A2.3 | 35 |  | 18 |  |  |  |  |  |  |  |  |  |  |  |  |  |
| A3.1 | 11 |  | 47 |  |  |  |  |  |  |  |  |  |  |  |  |  |
| A3.2 | 10 |  | 150 |  |  | 1 |  |  |  |  |  |  |  |  |  |  |
| A3.3 | 23 |  | 186 |  |  |  |  |  |  |  |  |  |  |  |  |  |
| B1.1 | 91 |  | 22 | 20 |  |  |  | 59 |  |  |  | 4 |  |  |  |  |
| B1.2 | 18 |  | 5 | 2 |  |  |  | 7 | 3 |  |  |  |  |  |  |  |
| B1.3 | 16 |  | 3 | 3 |  |  |  | 31 |  |  |  | 1 |  |  |  |  |
| B2.1 | 22 |  | 10 | 1 |  |  |  | 30 |  |  |  |  |  |  |  |  |
| B2.2 | 1 |  | 2 | 1 |  |  |  | 26 | 2 |  |  | 5 |  |  |  |  |
| B2.3 | 5 |  | 1 | 2 |  |  |  | 54 | 7 |  |  | 16 |  |  |  |  |
| B3.1 |  |  |  |  |  | 1 |  |  |  |  |  |  |  |  |  |  |
| B3.2 | 1 |  | 2 |  |  |  |  | 1 | 1 | 3 |  |  | 1 |  |  |  |
| B3.3 |  |  | 1 |  |  | 1 |  |  |  |  | 1 |  |  |  |  |  |
| C1.1 | 3 |  | 1 |  |  |  |  | 1 |  |  |  |  |  |  |  |  |
| C1.2 | 2 |  |  |  |  |  |  |  |  |  |  | 1 |  |  |  | 1 |
| C1.3 | 7 | 1 |  |  |  |  |  |  |  |  |  |  |  |  |  |  |
| C2.1 | 1 |  |  |  |  |  |  | 20 |  |  |  | 45 |  |  | 1 | 1 |
| C2.2 | 1 | 4 | 1 |  |  |  |  | 14 | 1 |  |  | 7 |  |  | 1 |  |
| C2.3 |  | 3 | 1 |  |  |  |  | 24 | 1 |  |  | 54 | 1 |  | 16 |  |
| C3.1 |  |  |  |  |  | 1 | 1 |  |  |  |  |  |  | 1 |  |  |
| C3.2 |  | 1 |  |  |  |  |  | 3 |  | 3 | 11 | 9 | 9 | 1 | 1 |  |
| C3.3 |  |  |  |  |  |  |  |  |  | 1 | 1 | 1 | 18 | 5 | 10 |  |

Blue boxes: number of combinations found in unselected populations in two registry studies (n = 1807)

Grey boxes: other combinations found in studies with selected patients (n = 723)
